# Supplementary material for: Flat-Band Lasing in Silicon Waveguide-Integrated Metasurfaces
Source: ACS Photonics. 2025 Mar 7;12(3):1570–8. doi: 10.1021/acsphotonics.4c02332 (PMC11926949; doi:10.1021/acsphotonics.4c02332)
Supplement: Supplementary file 1 — ph4c02332_si_001.pdf [file ph4c02332_si_001.pdf]

Supporting Information: Flat-band lasing in silicon waveguide-integrated metasurfaces

Sioneh Eyvazi, Evgeny A. Mamonov, Rebecca Heilmann, Javier Cuerda, and Päivi Törmä*

Department of Applied Physics, Aalto University School of Science, P.O. Box 15100, Aalto, FI-00076, Finland

E-mail: paivi.torma@aalto.fi

S1. Electric field distribution for TE_w flat mode

S2. Correlation between the flat band and dark modes in different planes of incidence

S3. Polarization vorticity feature in real-space images

S4. Characteristics of polarization vorticity in lasing emission within energy-momentum spectra

S5. Polarization vorticity feature in an array hosting extended flat mode in momentum-space

S6. Sample fabrication process

The file contains 6 pages and 6 figures.

S1 Electric field distribution for TE_w flat mode

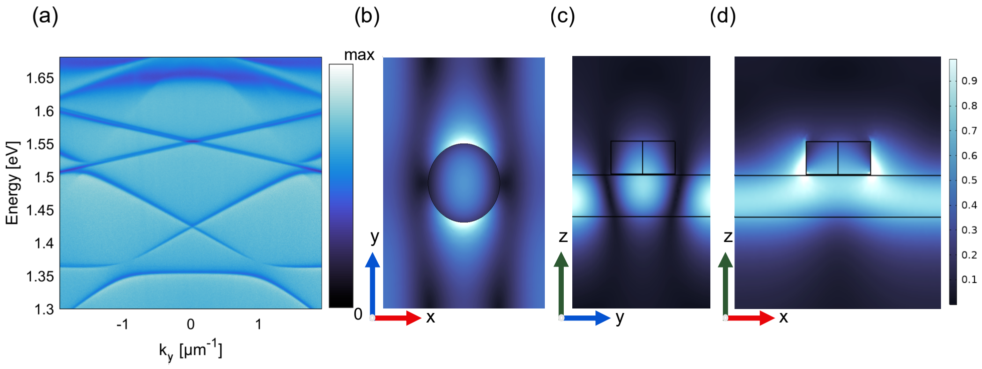

Figure S1: TE_w flat mode field distribution. (a) Transmission spectrum of an array with periodicities 340 nm in the x direction and 480 nm in the y direction. TE_w flat mode appeared at 1.35 eV. Electric field distribution of the flat mode at normal incidence ($k_y = 0$), (b) in xy, (c) zy, and (d) zx plane of view. The electric field intensity is mainly within the waveguide layer and does not reach much above it (where the gain medium would be).

S2 Correlation between flat band and dark modes in different planes of incidence

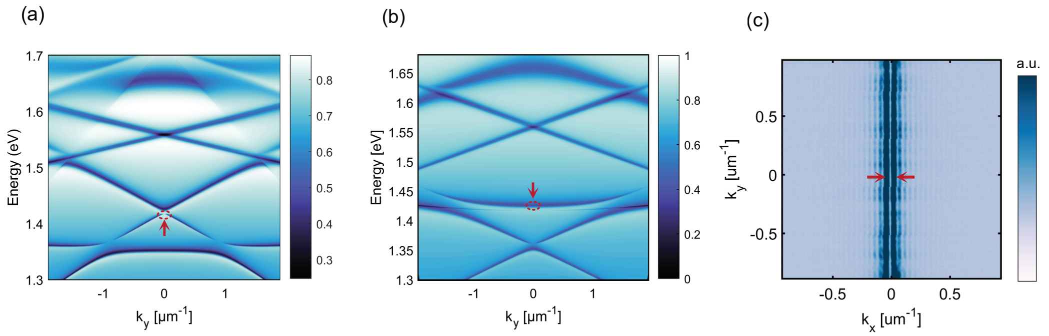

Figure S2: Correlation between the flat band and dark modes via RCWA simulation in different planes of incidence. RCWA simulation for arrays with periodicities (a) 340 and 480 nm along the x and y direction, respectively, corresponding to the xz plane of incidence. (b) 480 and 340 nm along the x and y direction, respectively, corresponding to the yz plane of the incident. (c) 2D momentum-space image of flat band lasing radiation at $\mathrm{TE_e}$ polarization. The observed gap along the y direction at $k_x = 0$ (marked by two arrows) arises from the dark mode at $k_y = 0$ (denoted by a red circle) within the xz plane of incidence (indicated by a red circle in (a)) which corresponds to the same energy position of the flat band mode in yz plane of the incidence (marked by a red circle in (b)).

S3 Polarization vorticity feature in real-space images

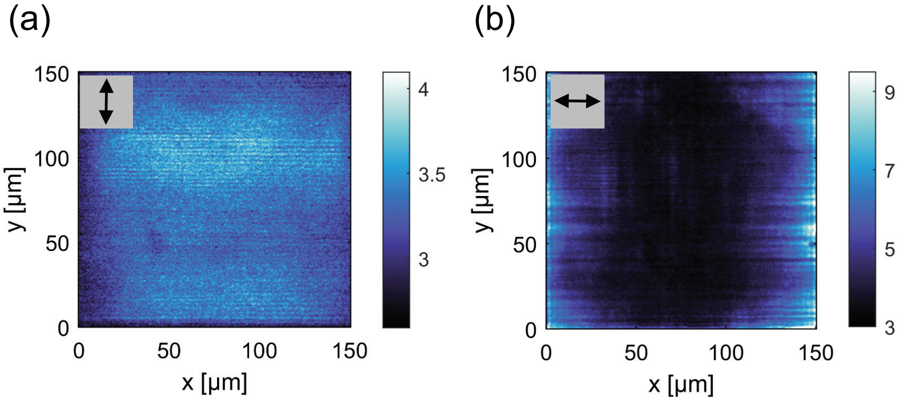

Figure S3: Real space images of lasing emission under different polarization filters for (a) TM_e (vertical arrow) and (b) TE_e (horizontal arrow) polarization correspond to a sample with periodicities of 480 nm and 340 nm in x and y direction, respectively. The pump fluence is 0.098 mJ/cm^2.

S4 Characteristics of polarization vorticity in lasing emission within energy-momentum spectra

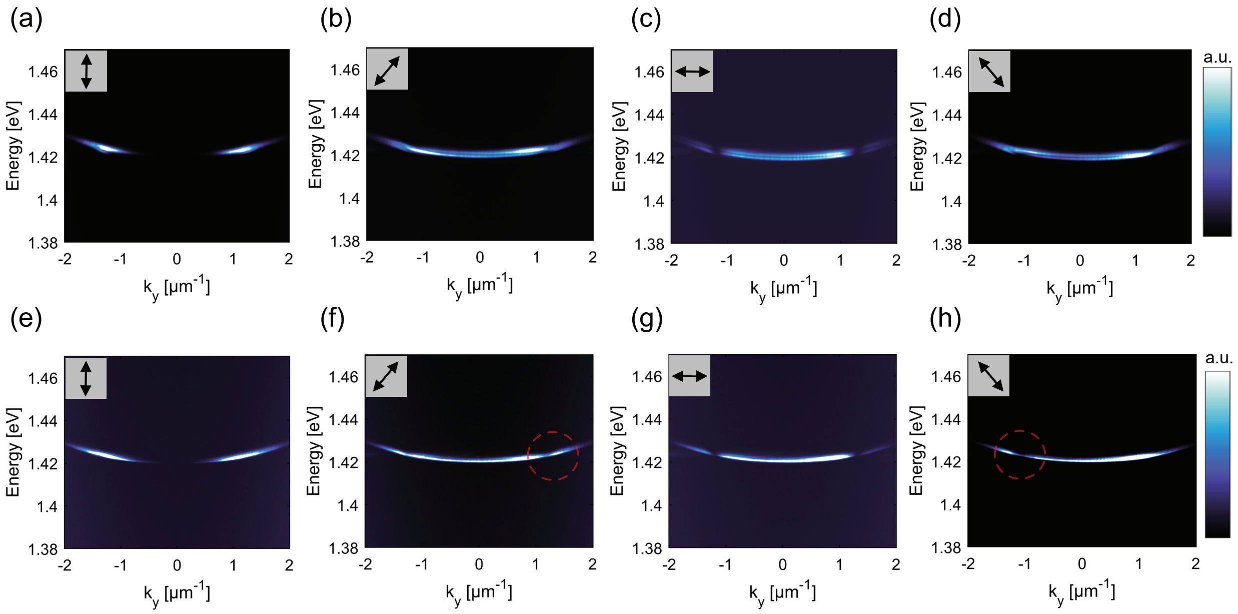

Figure S4: Polarization vorticity feature. Lasing emission in energy-momentum spectra in the presence of the gain medium under pump fluence of 0.098 mJ/cm^2, characterized by different polarization filters. The array has periodicities of 480 nm and 340 nm in the x and y directions, respectively. (a)-(d) displays lasing emission from the array when the spectrometer slit size is sufficiently wide to capture the entire k-space pattern depicted in panels (i-l) of Figure 4. (e)-(h) illustrate lasing emission slightly away from the Γ-point within the region of $k_x > 0$ (see the 2D k-space images presented in Figure 4i-l).

S5 Polarization vorticity feature in an array hosting extended flat mode in momentum-space

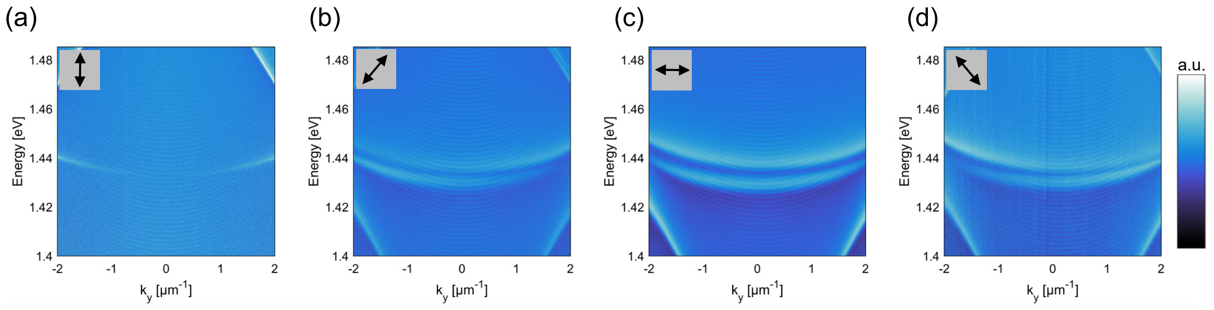

Figure S5: Polarization vorticity feature. (a)-(d) Energy-momentum dispersion measurements for any array with periodicities 475 and 350 nm along the x and y direction, respectively. The array is excited by a wide-range spectrum source for different analyzed polarization denoted by arrows. There is a clear asymmetry in the images for the two diagonal polarizations, demonstrating polarization vorticity.

S6 Sample fabrication process

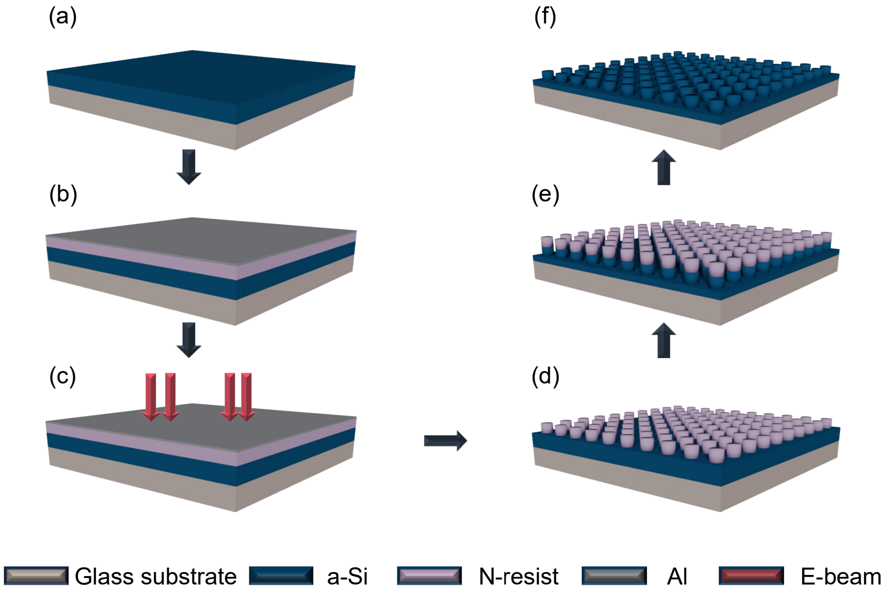

Figure S6: Fabrication process. (a) A thin 85 nm thick $\alpha-\mathrm{Si}$ is deposited on the cleaned borosilicate glass slide through the plasma-enhanced chemical vapor deposition (PECVD) method. (b) Negative electron beam resist is spin-coated on the sample. A thin conductive layer was deposited to prepare the sample for electron beam lithography (EBL). (c) The pattern is transferred to the sample by EBL. (d) the conductive layer is etched, and the resist is developed. (e) The sample is partially etched where the resist acts as a mask by the reactive-ion etching technique (RIE). (f) The oxygen-ashing process removes the resist.
